# Markers of neuroinflammation in the CSF of patients with difficult to treat psychiatric disease

**DOI:** 10.3389/fpsyt.2026.1665447

**Published:** 2026-05-04

**Authors:** Jocelyn X. Jiang, Artur Shvetcov, Nicole Fewings, Prudence Gatt, Suat Dervish, Justin Y Garber, Matthew Silsby, Alessandro F Fois, Stephen Duma, Sushil Bandodkar, Sudarshini Ramanathan, Andrew Bleasel, Bryne John, Ian B Hickie, Elizabeth M Scott, Anthony Harris, Ming-Wei Lin, Caitlin A. Finney, David A. Brown

**Affiliations:** 1 Department of Immunopathology, NSW Health Pathology-Institute of Clinical Pathology and Medical Research (ICMPR), Westmead Hospital, Westmead, NSW, Australia; 2 Sydney Medical School, University of Sydney, Sydney, NSW, Australia; 3 Department of Immunology, Blacktown Hospital, Blacktown, NSW, Australia; 4 Douglass Hanly Moir Pathology, Sydney, NSW, Australia; 5 Blacktown Clinical School, Western Sydney University, Blacktown, NSW, Australia; 6 Centre for Immunology and Allergy Research, The Westmead Institute for Medical Research, Westmead, NSW, Australia; 7 Department of Psychological Medicine, Sydney Children’s Hospitals Network, Sydney, NSW, Australia; 8 Brain Dynamics Centre, The Westmead Institute for Medical Research, Westmead, NSW, Australia; 9 Westmead Research Hub, The Westmead Institute for Medical Research, Westmead, NSW, Australia; 10 Department of Neurology, Westmead Hospital, Westmead, NSW, Australia; 11 Department of Clinical Biochemistry, The Children’s Hospital at Westmead, Westmead, NSW, Australia; 12 Department of Neurology, Concord Hospital, Concord, NSW, Australia; 13 Translational Neuroimmunology Group, Kids Neuroscience Centre and ANZAC Research Institute, Sydney Medical School, Faculty of Medicine and Health, University of Sydney, Sydney, NSW, Australia; 14 Department of Anaesthetics, Westmead Hospital, Westmead, NSW, Australia; 15 Brain and Mind Centre, University of Sydney, Sydney, NSW, Australia; 16 Prevention Early Intervention and Recovery Service, Western Sydney Local Health District Mental Health Service, Westmead, NSW, Australia; 17 Specialty of Psychiatry, Sydney Medical School, University of Sydney, Sydney, NSW, Australia; 18 School of Medical Sciences, University of Sydney, Sydney, NSW, Australia

**Keywords:** atypical psychiatric syndromes, CSF, cytokine, neuroinflammation, treatment resistance

## Abstract

**Introduction:**

The immune system is recognized as participating in the pathophysiology of psychiatric disease and there is renewed interest in identifying biomarkers of this immune activation.

**Methods:**

We measured serum and cerebral spinal fluid (CSF) autoantibodies with other routine and novel markers of neuroinflammation, including CSF cytokines in patients with atypical psychiatric presentations of both psychotic and mood disorders (n=35). Their markers were compared with cohorts of non-inflammatory neurological disease (NIND) controls (n=18), patients with central nervous system (CNS) viral infection (n=22) and autoimmune encephalitis (AE; n=40).

**Results:**

The most common autoantibody detected in the serum of patients with psychiatric disease were anti-nuclear antibodies followed by thyroid autoantibodies. Few atypical psychiatric patients had abnormal conventional CSF markers of neuroinflammation (pleocytosis, oligoclonal bands, abnormal CSF IgG: albumin ratio). Further analysis of CSF revealed elevation of ITAC/CXCL11 in the psychiatric cohort. TARC/CCL17 was lower in the psychiatric cohort compared to other groups in a random-effects multinomial model, despite no significant differences on univariate analysis. When the values of CSF cytokines were examined in individual patients, six patients (17%) had at least one CSF cytokine greater than four standard deviations above the mean of the NIND cohort group. Extensive serological evaluation revised the diagnoses of six (17%) of our psychiatric group, and these patients’ showed improvement with immunosuppression.

**Conclusion:**

Our results suggest a subset of people with atypical psychiatric disease may have a predominant immune contribution. This highlights the need for reevaluation and further consideration of differential diagnosis where patient presentations are not clinically typical, do not respond to conventional psychotropic treatment, or if other risk factors for autoimmunity are present.

## Introduction

Psychiatric disorders are associated with substantial morbidity and mortality (1), particularly in cases of treatment resistance, and current pharmacological therapies carry significant adverse effects (2, 3). A major barrier to improving outcomes is the incomplete understanding of the underlying pathophysiological mechanisms, which are likely diverse. Increasing evidence suggests that autoimmune and inflammatory processes may contribute to psychiatric disease, either through systemic or CNS signalling affecting the brain, or via direct immune-mediated mechanisms, as exemplified by autoimmune encephalitis (4).

Autoimmune encephalitis frequently presents with prominent psychiatric features, including psychosis and mood disturbance, although isolated psychiatric presentations occur in a minority of cases (5). An acute or subacute onset of symptoms is a key clinical criterion prompting consideration of autoimmune encephalitis (6). However, the growing recognition of this condition has renewed interest in inflammation and immune activation as potential pathogenic mechanisms underlying some primary psychiatric disorders (7, 8).

Associations between immune activation and psychiatric disease are well established. Infections are associated with an increased risk of subsequent psychiatric diagnoses, including viral pathogens such as cytomegalovirus (9) and toxoplasmosis (10), as well as sepsis and other bacterial infections (11–13). Maternal infection during pregnancy similarly increases the risk of psychiatric illness in offspring (14–16). In addition, autoimmune diseases are overrepresented among patients with bipolar disorder and schizophrenia (12, 13). Further, stress and trauma: recognized as risk factors for the development of psychiatric illnesses, are also associated with neuroinflammatory changes, which are described in both animal models (17), and patients (18). Postmortem brain sections of some patients with a diagnosis of schizophrenia have significant lymphocytic infiltrates (19, 20), perhaps indicating a predominant immune contribution or *forme fruste* of autoimmune encephalitis.

Collectively, these findings have informed a recent international consensus statement outlining clinical criteria for psychiatric disorders with a possible autoimmune aetiology (21). Despite this, there are few validated biomarkers capable of reliably identifying patients in whom autoimmune or inflammatory mechanisms are the primary drivers of psychiatric disease.

We recently reported a study investigating conventional and novel cerebrospinal fluid (CSF) markers of neuroinflammation in patients with antibody-negative autoimmune encephalitis, identifying IL-21 and IP10/CXCL10 as discriminators between autoimmune and viral encephalitis (22). Building on this work, we applied the same biomarker panel to a cohort of patients with atypical psychiatric presentations, to assess whether a subset demonstrated evidence of a predominantly autoimmune or inflammatory disease process (22).

## Methods

### Patient recruitment

Patients referred by treating psychiatrists for evaluation for a possible organic aetiology were assessed at a single quaternary referral centre in Western Sydney, Australia between 2016 and 2020. Referrals were guided by an international consensus framework identifying clinical “red flag” features suggestive of autoimmune psychiatric disease, including rapid progression, treatment resistance, personal or family history of autoimmunity, heightened psychotropic sensitivity, malignancy, or neurological features such as seizures, motor symptoms/movement disorder, reduced consciousness, aphasia, mutism, or dysarthria (21).

All patients underwent structured clinical assessment and extensive autoimmune serological testing. Those meeting red flag criteria underwent lumbar puncture, brain magnetic resonance imaging (MRI), and electroencephalography (EEG). The psychiatric cohort was compared with previously characterized cohorts of patients with autoimmune encephalitis (AE), non-inflammatory neurological disease (NIND), and viral infection (VI) controls (22). Ethical approval was obtained from the Western Sydney Local Health District Ethics Committee (LNR/16/WMED/192).

### Comparison groups

AE comparator data were derived from a previously reported cohort recruited over 18 months and classified by neurologists as high risk for autoimmune encephalitis using Graus criteria, with or without detectable neuronal autoantibodies (6, 22). NIND controls included patients undergoing lumbar puncture for other routine indications (e.g. idiopathic intracranial hypertension, normal pressure hydrocephalus, and perioperative spinal anaesthesia). VI controls consisted of de-identified CSF samples with viral infection confirmed by PCR or appropriate microbiological testing.

### Sample collection and laboratory investigations

CSF samples were collected in standard 10-mL tubes; aliquots for cytokine analysis were stored at −70 °C. Up to 1 mL of CSF was collected in RPMI medium for flow cytometric analysis. Assays for CSF light chains and CSF cytokines were batched for analysis to minimise analytical variation.

All investigations were performed at New South Wales Health Pathology, Institute for Clinical Pathology and Medical Research (ICPMR-Westmead, Australia), unless otherwise specified. Conventional serum and CSF investigations for autoimmune encephalitis have been reported previously (22) (oligoclonal bands, neopterin, detection of neuronal and limbic encephalitis antibodies, CSF microscopy, culture and protein levels and PCR for viral infections), with assay details provided in **Supplementary Methods**.

The following routinely available diagnostic tests were also performed on serum on patients with psychiatric disease: anti-thyroid peroxidase antibody and thyroglobulin antibody, ANA, ANCA, ENA dsDNA coeliac autoantibodies, EPG/IFE ESR, liver autoantibodies by indirect immunofluorescence), anti-GAD antibodies anti IA2 and anti-ZNT8 antibodies, IgG, IgA, IgM,C3, C4, IgG subclasses as well as voltage-gated potassium antibodies (VGKC) and voltage gated calcium channel antibodies (VGCC). CSF of psychiatric patients also underwent examination by flow cytometry.

All conventional assays followed standard clinical and diagnostic laboratory protocols. Due to limited CSF volumes in NIND and VI cohorts, analyses prioritized novel biomarkers lacking established reference ranges, including CSF cytokines.

### Cytokine analysis

CSF cytokines were performed using a bead-based multiplex Luminex (Milliplex™) assay on a research base only. Cytokines tested were: IFN-γ, ITAC (CXCL11), IL-12p70, TNF-α, CXCL9, CXCL10/IP-10, IL-13, IL-4, IL-5, TARC (CCL17), Eotaxin, IL-17a, IL-6, IL-8, L-1b, IL-21, IL-2, IL-23, IL-7, IL-10, BCA-1 (CXCL13), GMCSF and GCSF. Further assay details are available in the **Supplementary Methods**.

### Collection of clinical information

Clinical details in the psychiatric cohort, AE patients and NIND and cohorts were collected during patient assessment, treating clinicians and verified through medical records. These included any concurrent medications included medication and medication failures, adverse effects, psychiatric and cognitive symptoms, seizures, headaches, pain, paraesthesia, movement disorders, sleep disturbance, family or personal history of autoimmunity and past history of cancer. Additional clinical details were also collected for the purposes of interpretation of any positive diagnostic test results. Clinical data were unavailable for VI samples, which were provided de-identified.

### Statistical analysis

Statistical analysis was performed using StataMP v19 and R studio. Results that were lower than the limit of detection were designated as “0” for statistical analysis. Scatterplot figures were generated in GraphPad Prism v10. For scatterplots, values were log-transformed after adding 1 to all observations (log10[x + 1]). Heatmap plots were generated using scaled variables in R (v 3.6.3) and R Studio (v1.2.5033) using the “pheatmap” library. Principal component analyses were performed in R (v 3.6.3) and R Studio (1.2.5033) using libraries “factoextra” and “factominer”.

Group comparisons used Wilcoxon rank-sum or Kruskal–Wallis tests for continuous variables and Pearson’s chi-squared tests for categorical variables. To account for multiple testing, p-values were adjusted using the Bonferroni correction. After excluding variables for collinearity, we standardised the results of the cytokines using log normalisation and z-standardisation before analysing results in a random-effects multinomial logistic regression model, accounting for batch-to-batch variation via a shared random intercept.

## Results

### Cohort characteristics

Thirty-five patients with treatment-resistant psychiatric disease were included and compared with cohorts of NIND (n=18), VI (n=22), and AE (n=40) controls. Clinical characteristics are summarized in **Table 1**, with psychiatric symptom profiles detailed in **Table 2**.

**Table 1:** Clinical characteristics.

Group	N	Age (yrs) median (range)	M:F
Psychiatric Disease	35	20 (15-60)	1:4.8
Autoimmune Encephalitis	40	38.5 (15-73)	1:1.1
Non-inflammatory neurological disease	18	45 (17-81)	1:3.5

**Table 2:** Clinical features of included psychiatric patients.

Characteristic	All patients
Psychiatric Diagnosis n (%)	
Depression	10 (29%)
Mania/Bipolar disorder	6 (17%)
Chronic non-affective psychosis/schizophrenia	7 (20%)
First episode psychosis	4 (11%)
Multiple psychiatric diagnosis	8 (23%)
Other Symptoms n (%)	
Sleep Disturbance	4 (11%)
Seizures (any previous history)	5 (15%)
Pseudo seizures	2 (6%)
Fatigue	15 (43%)
Cognitive Issues	10 (29%)
Movement Disorders	1 (2%)
Infectious Prodrome	0 (0%)
Paraesthesia	3 (9%)
Chronic pain	11 (31%)
Other Associated Features n (%)	
History of Autoimmunity	18 (51%)
Family history of Autoimmunity	11 (31%)
Treatment Effect	
Failed at least 1 psychiatric medication	27 (77%)
Failed at least 2 psychiatric medications	20 (57%)

The psychiatric cohort comprised 29 females and 6 males (median age 20 years, range 15–60). Primary presentations included mood disorders (n=21) and psychotic symptoms (n=14). A personal history of autoimmune disease was present in 18 patients, and a family history in 11. Additional accompanying physical symptoms included fatigue (15/35), chronic pain (11/35), cognitive symptoms (10/35), prior seizures or non-epileptic seizures (6/35), sleep disturbance (4/35), and paraesthesia (3/35).

The NIND cohort consisted of 14 female and four males with a median age of 45.5 and age range of 17-81. Ten of these patients had a diagnosis of idiopathic intracranial hypertension (IIH), two had a diagnosis of primary headache disorder, two had normal pressure hydrocephalus (NPH) and four patients had CSF samples taken prior to administration of spinal anaesthesia.

In the AE cohort there were 40 patients in total: 13 who were antibody positive and 27 who were antibody negative. There were 21 females and 19 males. The median age was 38.5 with an age range of 15-73. No clinical data was available for the 21 de-identified VI controls (22).

#### Serum markers of inflammation and autoimmunity

Serum inflammatory markers and autoantibodies were available only for the psychiatric cohort. Antinuclear antibodies (ANA) were the most frequently detected autoantibody, present in 23/32 (72%) patients, however these were predominantly (n=20/23) at low titres (1:80–1:160) and without associated extractable nuclear antibodies (ENA) or double stranded DNA (dsDNA) antibodies. The most common ANA pattern was speckled (19/23) followed by homogenous (2/23), nucleolar (1/23) and speckled and mitotic spindle apparatus (1/23). Two patients had mid–high titre ANA (1:640) without clinical features of connective tissue disease, while one patient had a high-titre ANA (1:2560) with anti-SSA/Ro60 positivity.

Thyroid autoantibodies were detected in 7/35 (20%) patients for thyroid peroxidase antibodies and 3/35 (8%) for thyroglobulin antibodies; only two had biochemical thyroid dysfunction. One patient had a positive c-ANCA but without myeloperoxidase (MPO) or proteinase 3 (PR3) antibody detected and no clinical features of ANCA-vasculitis. A non-specific ANCA pattern was detected in 7/35 patients (20%), again without associated MPO or PR3 antibodies. Single cases of positive coeliac serology and borderline myositis-associated antibodies were identified. No patients had anti-double stranded DNA antibody, liver autoantibodies, or other diabetes associated antibodies. One of 35 patients had positive serum voltage gated calcium channel (VGCC) without clinical evidence of Lambert-Eaton myasthenic syndrome or malignancy. No serum onconeural or limbic encephalitis antibodies were detected.

Markers of non-specific immune activation were infrequent: erythrocyte sedimentation rate (ESR) was elevated in 3/33 (9%) patients, one patient had elevated immunoglobulin G (IgG), one had polyclonal hypergammaglobulinemia on electrophoresis (although serum IgG was within normal limits), and one had serum oligoclonal bands.

### Conventional CSF markers

Conventional CSF markers were available for all psychiatric and AE patients, but only partially for NIND controls (13/18 for CSF protein quantitation and cell count; 8/18 for oligoclonal bands; 9/18 for neopterin) (**Table 3**).

**Table 3:** Conventional Serum and CSF results.

Investigation finding	Psychiatric disease (N = 35)	Non-inflammatory control (N = 9)	Unadjusted P-value (psychiatric disease vs NIND)	Bonferroni adjusted P value (psychiatric disease vs NIND)	Autoimmune encephalitis (N = 40)	Unadjusted P-value (psychiatric disease vs AE)	Bonferroni adjusted P value (psychiatric disease vs AE)
Elevated CSF Protein	5 (14%)	3 (3%)	0.47	1	24 (60%)	0.02	0.6
CSF Mononuclear >5	1 (3%)	1 (11%)	0.46	1	22 (55%)	0.002	0.06
CSF Oligoclonal bands	4 (11%)	0 (0%)	0.3	1	15 (38%)	0.008	0.2

One in the NIND had CSF pleocytosis (6 mononuclear cells); this patient had a diagnosis of IIH. Three had raised CSF protein (>0.45g/L): two with NPH and one with IIH. Five (56%) patients had evidence of raised neopterin (>20nmol/L). Three of these patients had a diagnosis of idiopathic intracranial hypertension and two of these had a diagnosis of NPH. One of the patients with NPH had a shunt at the time of lumbar puncture. No NIND patients demonstrated CSF-restricted oligoclonal bands.

Of the psychiatric cohort, 1/35 (2%) of patients had evidence of CSF pleocytosis (mononuclear cells >5 cells on CSF microscopy), 4/35 (11%) had CSF specific oligoclonal bands, 5/35 (14%) had elevated CSF protein (>0.45g/L) and 12/35 (34%) had raised CSF neopterin. These findings did not differ significantly from the NIND cohort.

In contrast, the AE cohort demonstrated 15/40 (38%) patients with elevated CSF protein, 12/40 (30%) patients with pleocytosis, 15/40 (38%) patients with oligoclonal bands and 29/35 (72%) patients with elevated neopterin. After Bonferroni correction, elevated neopterin remained the only statistically significant difference between AE and psychiatric cohorts (χ²=17.01, p=0.003).

CSF IgG, albumin and IgG:albumin ratio data were available in a subset of our cohorts due to preanalytical sample loss. A raised ratio was observed in 1/22 psychiatric patients, compared with 10/19 AE patients and 2/5 NIND patients but these differences were not significant after Bonferroni correction. Of the 2 patients in the NIND group with raised IgG: albumin ratio, one had IIH and the other NPH, but the CSF was taken prior to insertion of any shunts. Reibergram (23) analysis was performed on patients with available CSF IgG and albumin data. This demonstrated normal profiles in all psychiatric patients, while two AE patients showed evidence of blood–brain barrier dysfunction and two had evidence for intrathecal IgG synthesis and one patient had both abnormalities. One of the NIND cohort also had Reibergram evidence of blood-brain barrier dysfunction; he had NPH and a shunt *in situ* at the time of the lumbar puncture.

No psychiatric patients had CSF onconeural or limbic encephalitis antibodies or abnormal neuronal immunofluorescence.

### CSF beta-2-microglobulin

CSF beta-2-microglobulin did not differ between AE and NIND cohorts (z=−0.53, p=0.6). There was a trend for the level of CSF beta2-microglobulin to be lower in the psychiatric cohort compared to NIND, but this was not significant after correction for multiple comparisons (median percentile difference 0.45, 95%CI 0.15-0.81, Mann-Whitney z=2.9 p=0.12). Levels were significantly lower in the psychiatric cohort compared with AE (median percentile difference 0.54, 95% CI 0.22–0.93; z=3.58, p=0.009).

### Flow cytometry results

CSF flow cytometric data was only obtained on 25/35 patients in the psychiatric disease cohort, as some CSF was collected without Roswell Park Memorial Institute media and viable cells for analysis could not be obtained. Twelve samples had low cell counts and could not be interpreted. Eight patients demonstrated reduced CD4:CD8 ratios, one had an increased ratio, and three had detectable B cells (<1%), consistent with reported normal CSF ranges (24, 25).

### CSF cytokines

**Figure 1:**
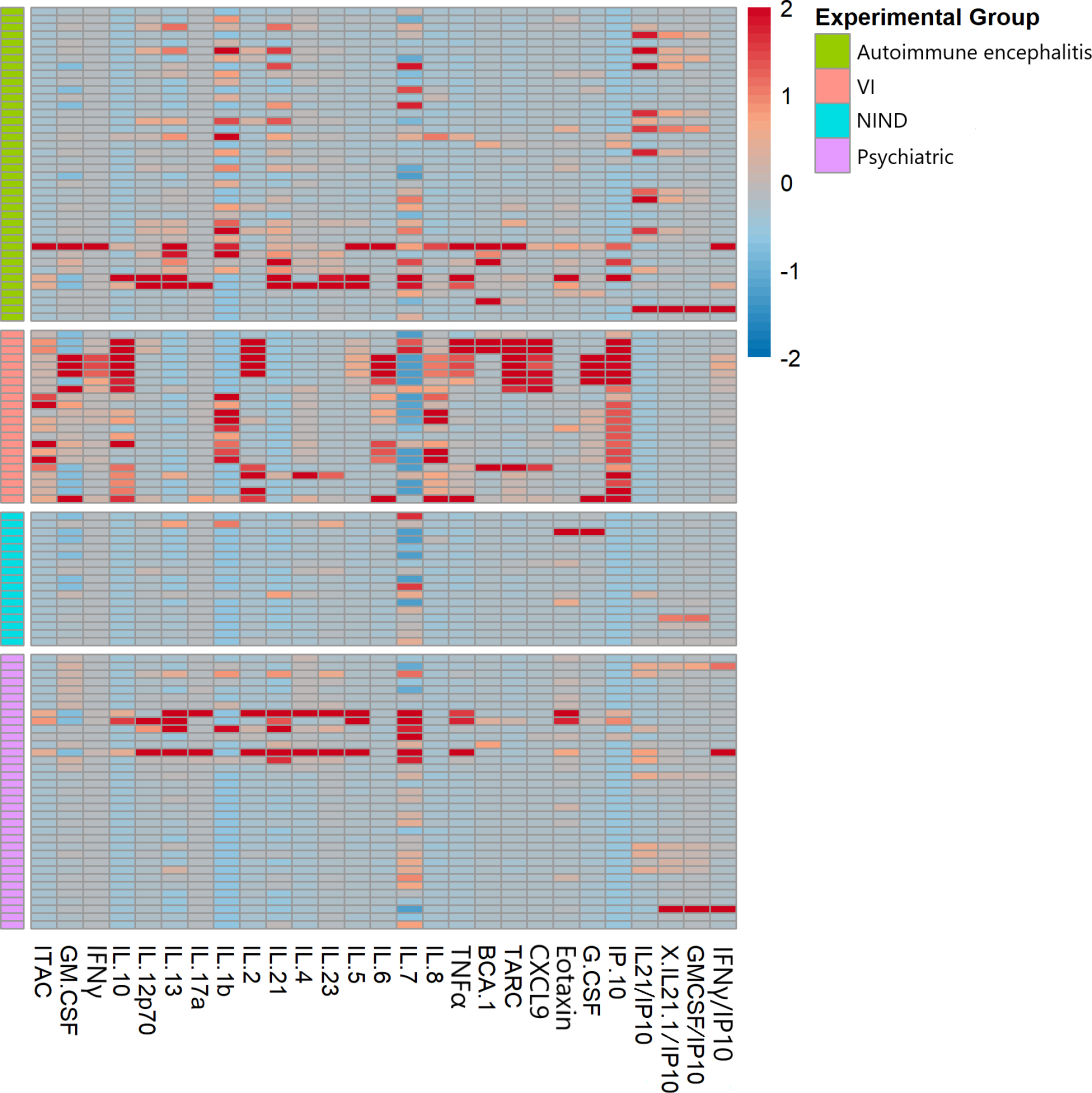
Heat map showing that psychiatric and autoimmune encephalitis cohorts had subsets of patients with higher CSF cytokine expression, while VI controls displayed more uniform cytokine elevation in a different pattern.

Heatmap analysis demonstrated high cytokine expression in the VI cohort, consistent with prior findings (22). Cytokine expression in the AE and psychiatric cohorts was variable with differently elevated cytokines compared with VI (**Figure 1**).

Cytokines found to be elevated on univariate analysis are summarised in **Table 4**. After Bonferroni correction, the cytokine IFN-γ was significantly elevated in the psychiatric cohort compared to NIND (median percentile difference 0.11, 95%CI 0.08-0.21 Mann-Whitney z=-3.5 p=0.02). Compared with VI controls, psychiatric patients had higher levels of IL-13 (median percentile difference 0.8, 95%CI 0.34-1.26 Mann-Whitney z=-3.642 p=0.009), IL2-1 (median percentile difference 0.65, 95%CI 0.34-1.01 Mann-Whitney z=-4.4 p=0.003), IL-7 (median percentile difference 9.7, 95%CI 5.85-13.4 Mann-Whitney z=-3.789 p=0.006) and IL23 (median percentile difference 8.4, 95%CI Mann-Whitney 3.936-9.89 z=-5.104; p=0.003). The cytokines ITAC, IFN-γ, IL-10, IL-6, IL-8, TNF-α, TARC, BCA-1, CXCL9, GCSF and IP10 were significantly higher in the VI cohorts (**Table 4**).

**Table 4:** Significant cytokines in univariate analysis.

Cytokine	Median percentile difference	95% CI	Man-Whitney z score	Unadjusted P-value	Bonferroni Adjusted P-value	Elevated in
Psychiatric vs NIND						
ITAC	1.33	0.33-2.37	-3.145	0.0017	0.05	Psychiatric
GMCSF	0.14	0.009-0.37	-2.479	0.013	0.4	Psychiatric
IFN-γ	0.11	0.08-0.21	-3.5	0.0005	0.015	Psychiatric
IL-12p70	0.06	0-0.22	-2.365	0.018	0.6	Psychiatric
IL-13	0.45	0-1.08	-1.97	0.049	1	Psychiatric
IL-21	0.45	0.16-0.88	-2.698	0.007	0.2	Psychiatric
IL-23	2.98	0-7.73	-2.072	0.038	1	Psychiatric
TNFα	0.32	0.09-0.53	-2.62	0.009	0.27	Psychiatric
Psychiatric vs VI						
IL-13	0.8	0.34-1.26	-3.642	0.0003	0.009	Psychiatric
IL-21	0.65	0.34-1.01	-4.4	<0.0001	0.003	Psychiatric
IL-7	9.7	5.85-13.4	-3.789	0.0002	0.006	Psychiatric
IL-23	8.4	3.94-9.89	-5.104	<0.0001	0.003	Psychiatric
ITAC	56.75	41.9-94.27	5.606	<0.0001	0.003	VI
IFN-γ	30.17	15.7-55.32	5.38	<0.0001	0.003	VI
IL-10	66.58	42.17-102.19	5.905	<0.0001	0.003	VI
IL-2	0.24	0-4.5	2.036	0.04	1	VI
IL-6	88.92	13.89-343.34	5.9	<0.0001	0.003	VI
IL-8	525.26	291.59-779.84	6.31	<0.0001	0.003	VI
TNFα	3.4	1.9-7.33	4.246	<0.0001	0.003	VI
TARC	3.01	-0.12-16.89	2.012	0.04	1	VI
BCA-1	3.43	1.72-5.3	4.409	<0.0001	0.003	VI
CXCL9	1129.16	785.85-13133.14	6.114	<0.0001	0.003	VI
GCSF	84.32	25.59-145.89	4.5	<0.0001	0.003	VI
IP-10	27659.24	26540.87-35850.77	6.3	<0.0001	0.003	VI
Psychiatric vs AE						
ITAC	1.02	0.92-2.14	-2.9	0.004	0.11	Psychiatric
IFN-γ	0.08	0-0.16	-2.812	0.005	0.2	Psychiatric
IL-1b	0.08	0-0.18	2.981	0.003	0.09	AE
IL4	0.51	0-1.9	2.142	0.03	0.9	AE
IL-10	0.9	0.09-1.1	2.69	0.007	0.2	AE
IL21	0.63	0-1.49	2.013	0.04	1	AE

**Figure 2:**
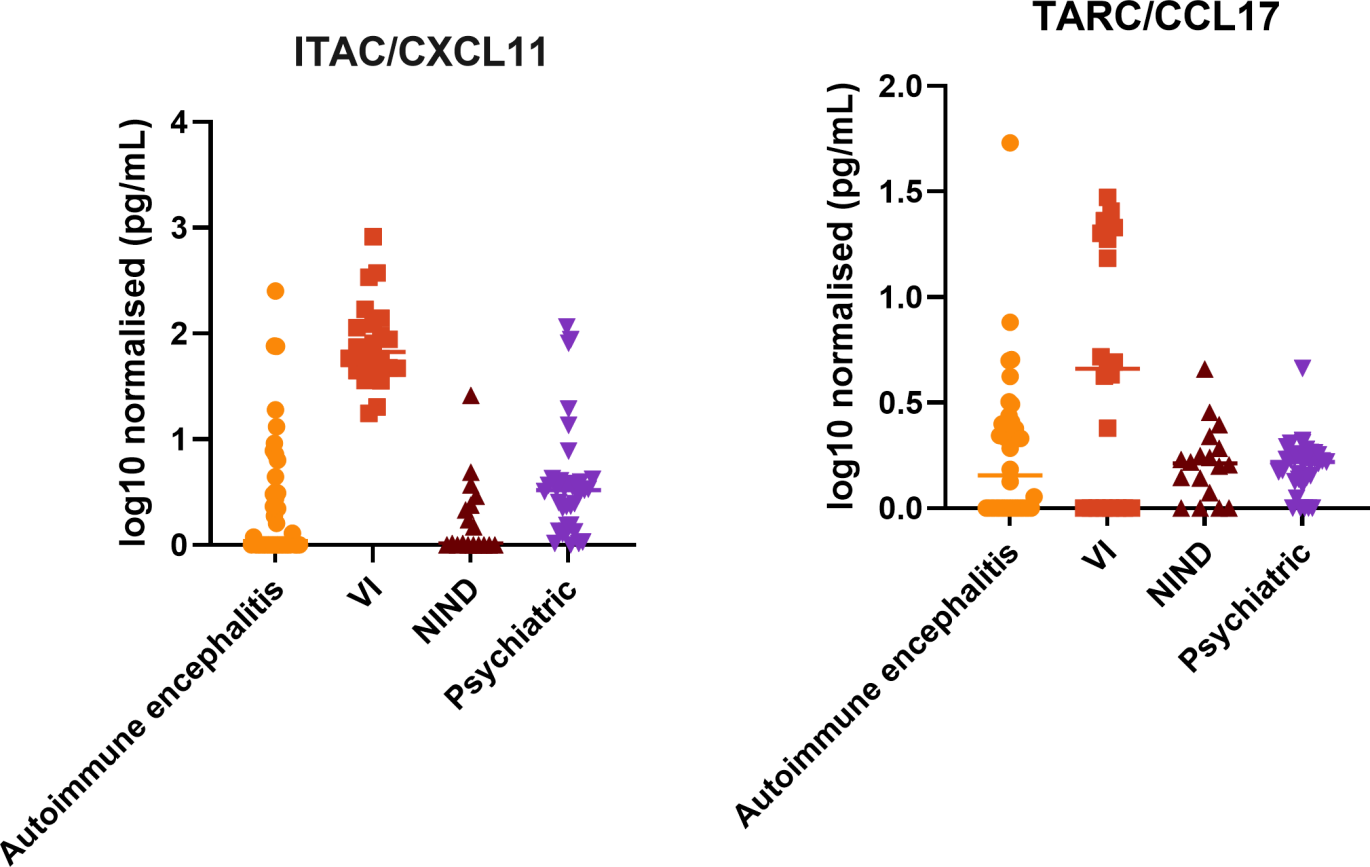
ITAC/CXCL11 and TARC contributed significantly to the multivariate model with batch number of testing included in the analysis. ITAC/CXCL11 was raised in the psychiatric cohort compared to both NIND and AE patients but was lower than levels in the VI (infection) cohort. Whilst TARC was not significantly different to comparator cohorts in the univariate analysis it did make a significant contribution to the model.

After analysing results in a random-effects, multinomial logistic regression model, the cytokines TARC and ITAC showed associations with the psychiatric cohort (**Figure 2**, **Table 5**). The cytokine ITAC was higher in the psychiatric cohort when compared with the NIND (Relative Risk Ratio (RRR)= 10.9 95%CI 2.2-52.8 z=-2.96 p=0.003) and AE cohorts (RRR = 5.5 95%CI 1.62-18.59 z=-2.74 p=0.006) but was lower in the psychiatric cohort when compared with the VI cohort (RRR = 0.21 95%CI 0.05-0.88z= 2.14 p=0.03).

**Table 5:** Significant cytokines in multivariate analysis when the psychiatric cohort is compared to a combined cohort of NIND, AE and viral controls in multinomial logistic regression model.

Cytokine	Relative Risk ratio	Standard error	Z-score	P-value	95% confidence interval
AE vs Psychiatric					
TARC/CCL17	0.058	18.11	2.74	0.006	0.0075-0.44
ITAC/CXCL11	5.5	0.11	-2.74	0.006	1.62-18.59
Viral Infection vs Psychiatric					
TARC/CCL17	0.077	13.01	2.54	0.01	0.01-0.55
ITAC/CXCL11	0.21	3.50	2.14	0.032	0.05-0.88
NIND vs Psychiatric					
TARC/CCL17	0.052	21.24	2.68	0.007	0.006-0.45
ITAC/CXCL11	10.9	0.74	-2.96	0.003	2.2-52.8

In univariate analysis and after Bonferroni correction, the only significant difference in TARC concentrations was elevation in the VI cohort compared with the psychiatric cohort. However, in random-effects multinomial logistic regression model, TARC was consistently lower in the psychiatric cohort (**Table 5**) when compared with the NIND (RRR = 0.052 95%CI 0.006-0.45 z= 2.68 p=0.007), AE (RRR = 0.058 95%CI 0.0075-0.44 p=0.006) and VI cohorts (RRR = 0.077 95%CI 0.01-0.55.00 z= 2.54 p=0.01). Although the confidence intervals were wide, indicating uncertainty in the magnitude of effect, the direction of association was consistent across comparator groups, suggesting TARC does provide independent discriminatory information for the psychiatric cohort after adjustment for correlated cytokines.

A subset of psychiatric patients (6/35, 17%) demonstrated marked cytokine elevation (>4 SD above NIND mean). Three of these patients had at least 2 cytokines elevated four standard deviations above the mean cytokine levels in NIND cohort. The elevated cytokines were a mix of T-cell related, B-cell related and pro inflammatory cytokines ITAC (n=1), IFN-γ (n=1), IL-10 (n=1), IL-12p70 (n=1), IL-17a (n=1) IL-1b (n=1), IL-2(n=2), IL-4 (n=2), IL-23 (n=1), IL-5 (n=2), TNF-α (n=1), BCA-1 (n=1), CXCL9 (n=1) and IP-10 (n=1).

This subgroup was clinically heterogeneous, comprising patients presenting with depression (n=1), bipolar disorder (n=3), or psychosis (n=2). Five were female and one male, with an age range of 16–30 years. A personal history of autoimmunity was present in three patients, and a family history in two; one patient had no personal or family history of autoimmunity. Two reported concurrent fatigue and three reported cognitive dysfunction.

Not all patients with markedly elevated CSF cytokines demonstrated abnormalities in conventional CSF markers of neuroinflammation: in two cases, routine CSF findings were entirely normal apart from neopterin. Among the remainder, abnormalities included CSF pleocytosis (n=1), CSF-restricted oligoclonal bands (n=1), and elevated CSF protein (n=3); notably, all patients in this subgroup had raised CSF neopterin (>20 ng/mL).

### Other investigations

Brain MRI was available for 28 psychiatric patients: 22 were normal and six showed minor non-specific white matter changes. EEG was performed in 22 patients; abnormalities included diffuse cerebral dysfunction (n=7), focal dysfunction (n=1), and generalized epileptiform activity (n=1). The patient with epileptiform activity did not have elevated CSF cytokines, positive serum autoantibodies and her CSF analysis showed no indication of abnormal routine markers of neuroinflammation. She had already been taking lamotrigine for her psychiatric disease and gabapentin for chronic pain at the time of EEG.

### PCA analysis

**Figure 3:**
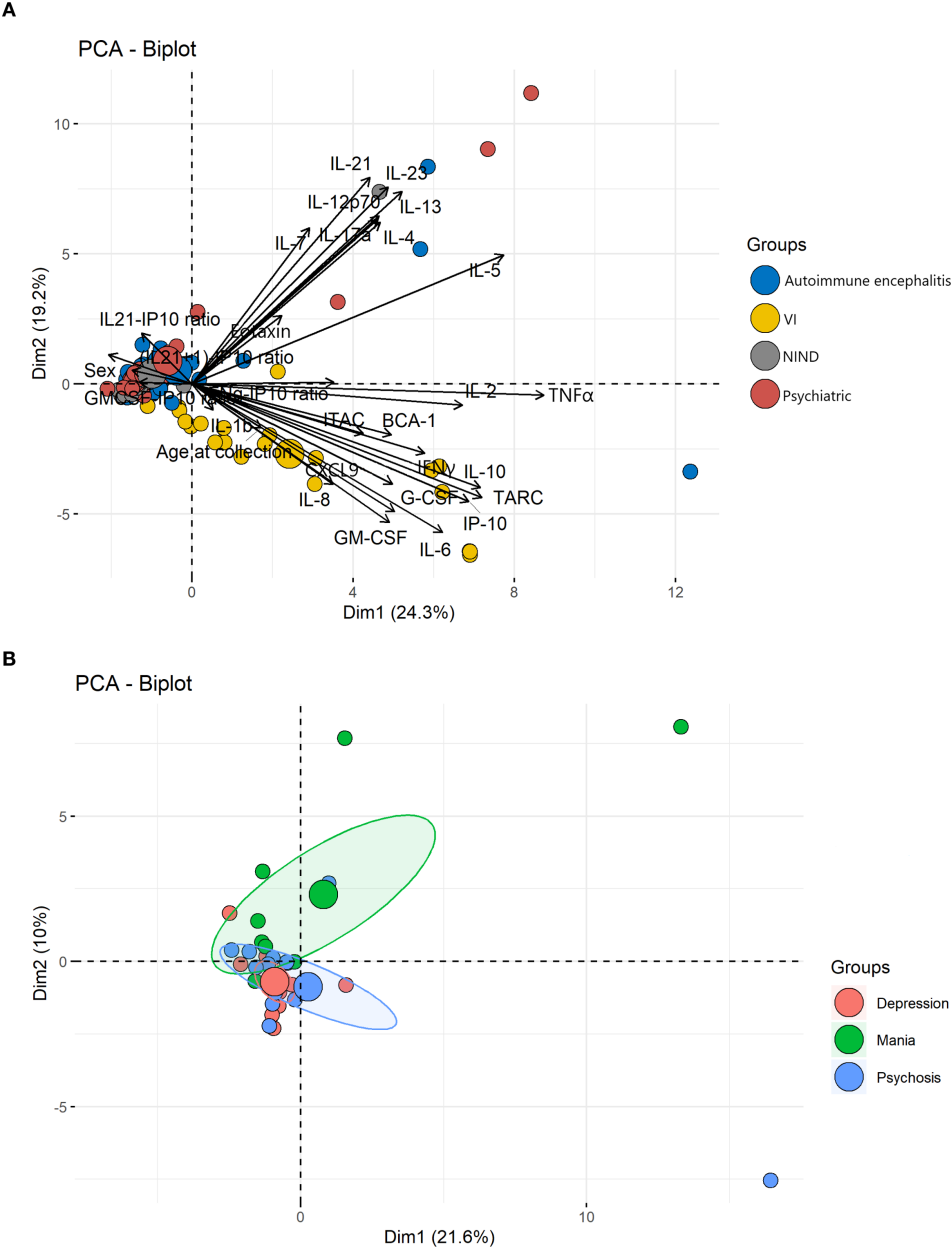
Principal component analysis (PCA) biplots differentially cluster diagnostic and symptom groups based on cytokine profile. **(A)** The PCA biplot compares diagnostic cohorts, showing each participant as a point coloured by their diagnostic group. Arrows indicate the direction and strength (loadings) of individual cytokines for each principal component. The axes represent the first two components, accounting for 24.3% (Dim1) and 19.2% (Dim2) of the total variance. The infectious cohort is distinct along Dim1 suggesting a unique global inflammatory profile. By contrast, autoimmune encephalitis, psychiatric, and non-inflammatory groups substantially overlap, indicating similar or mixed cytokine patterns rather than a single inflammatory response. **(B)** PCA is further divided by clinical presentation. Each patient is shown as a coloured point based on whether they presented with predominant mania, depression, or psychosis, while shaded ellipses reflect group dispersion. The first two principal components explain 21.6% (Dim1) and 10.0% (Dim2) of variance. Patients with mania show relative separation along Dim2, whereas those with depression and psychosis have considerable overlap. This suggests that manic patients may have a distinctive but varied cytokine profile, while depressive and psychotic presentations tend to share immune signatures.

PCA demonstrated distinct clustering of VI patients driven by CSF cytokine expression (**Figure 3a**). Substantial overlap was observed between psychiatric, NIND, and AE cohorts, consistent with biological heterogeneity. A subset of psychiatric patients with elevated cytokines clustered with AE cases. Patients with a history of mania showed partial separation from those with unipolar depression or psychosis (**Figure 3b**).

### Effect of psychotropic medications on CSF cytokines

Psychotropic medication use is summarized in **Table 6**. After correction for multiple comparisons, no cytokines differed by antipsychotic, lithium, or antiepileptic use. ITAC levels were lower in patients taking antidepressants (median difference 2.27 95% CI 0.97-6.75, Mann Whitney z=3.416, p=0.02). Medication use did not contribute significantly to the random-effects multinomial logistic regression model and further analysis for effect of dosage or duration of medication therapy was limited by low sample group numbers.

**Table 6:** Antipsychotic use in psychiatric patient cohort*.

Antipsychotic	Number of patients	Range of dose prescribed
Risperidone	2	0.5-3mg
Olanzapine	6	10-300mg
Aripiprazole	4	10-40mg
Quetiapine	7	25-200mg
Paliperidone	2	3-6mg
Haloperidol	1	3mg (patient recently ceased olanzepine15mg)
Clozapine	3	175-200mg
Amisulpride	1	400mg

*No dose data was available for 4 patients. They were prescribed aripiprazole (2), paliperidone (1) and quetiapine. Six patients were prescribed two concurrent antipsychotics. Of these for 2 patients the second antipsychotic was prescribed on a ‘taken when necessary’ basis.

### Change in diagnosis in a subset of our patients

Six psychiatric patients (17%) had their diagnosis revised to a defined autoimmune disorder following clinical reassessment and immunomodulatory treatment (**Table 7**). All demonstrated clinical improvement with disease-specific therapy and reduced psychotropic requirements.

**Table 7:** Patients where diagnosis changed.

Patient number	Age	Sex	Presenting features	Positive test results suggesting immune involvement	Treatment	Time from symptom onset to treatment response (months)	CSF cytokines increased?	New diagnosis	Treatment response
1	16	M	Treatment resistant depression and psychosis	High positive thyroperoxidase antibody Single band seen on CSF oligoclonal bands Increased CSF neopterin	Pulse methylprednisolone, IVIg, rituximab, mycophenolate	24	Y, cytokine IL1b greater than 4 SD above mean of NIND cohort	Hashimoto’s encephalopathy	Resolution of symptoms and eventual cessation of psychotropic medications and immunomodulatory therapy
2	30	F	New onset peripartum mania with short term memory loss. No previous psychiatric history. Developed a vasculitic rash with associated arthralgia and fevers.	Positive cryoglobulins 1:80 speckled ANA Increased CSF neopterin	Prednisolone and azathioprine with further consolidation of immunotherapy with rituximab post delivery	4	Y, cytokine CCL9 greater than 4 SD above mean of NIND cohort	Cryoglobulinaemic vasculitis	Resolution of mania with no long-term need for psychotropic medications. Relapse (Cryogloublins detected with monitoring) on next pregnancy aborted with pre-emptive rituximab therapy
3	16	F	New onset mania with prodromal period of short-term memory loss and brain fog. History of Hashimoto’s thyroiditis and new diagnosis of type 1 diabetes Strong family history of type 1 diabetes.	Non-specific VGKC antibody GAD65 serology (likely indicative of Type 1 diabetes) 1:2560 speckled ANA	mycophenolate	4	Y, cytokine BCA-1 greater than 4 SD above mean of NIND cohort	Hashiomoto’s encephalopathy Sjogren’s syndrome	Improvement in symptoms where patient was previously treatment resistant; antipsychotic dose was able to be lowered from maximal to low dose
4	20	F	Treatment resistant psychosis with paranoid delusions	Speckled 1:160 ANA Positive coeliac serology Positive CSF oligoclonal bands	Gluten free diet; mycophenolate started after symptoms returned	9	N	Coeliac disease	Resolution of symptoms
5	28	F	Treatment Resistant psychosis	VGCC channel antibodies Positive CSF neopterin	Pulse methylprednisolone, plasmapheresis, mycophenolate, IVIg	12	N	Autoimmune contribution to psychiatric disease	Functional improvement with return to work
6	17	F	Atypical mood and psychiatric symptoms with episodes of confusion and collapse. Problems with impulse thoughts and attempts at suicide and self-harm. Unilateral (right sided) piloerection, chronic pain, muscle spasm and severe headache. Generalised seizure activity was seen on prolonged video EEG. Previous EEG results prior to recruitment were non-specific.	Nil; MRI brain also normal	Prednisolone, mycophenolate, IVIg, Rituximab	5 years	N	Autoimmune encephalitis	Initial improvement in concentration, memory and hallucinations but an overall fluctuating clinical course

One patient’s diagnosis was revised to probable seronegative autoimmune encephalitis. This patient presented with long standing treatment resistant depression and psychosis with visual hallucinations. There was a history of seizures three years before presentation, attributed to a medication side effect and routine EEGs were previously normal. She also had physical symptoms: severe chronic pain, muscle spasms, migraines and unilateral piloerection, suggestive of focal autonomic seizures. A prolonged video recorded EEG identified generalized seizure activity. Her diagnosis was eventually revised 2 years after she was referred to our clinic, but after a trial of immunosuppression had commenced. She was treated with antiepileptics and immunosuppression including prednisolone, mycophenolate, intravenous immunoglobulin (IVIg) as well as Rituximab. She did report initial improvement with immunosuppression and IVIg: in her concentration, memory and hallucinations. Symptoms worsened just before her next IVIg dose was due. She has since had a fluctuating clinical course requiring ongoing antipsychotics including the introduction of low dose clozapine with ongoing immunomodulation.

Two patients were diagnosed with Hashimoto’s encephalopathy, with one patient having concomitant Sjogren’s syndrome. One patient had cryoglobulinemic vasculitis. Another patient had likely autoimmune contribution to psychiatric disease and improvement in functional ability (able to return to work), after a trial of Rituximab and mycophenolate. This patient had positive VGCC in serum, with no evidence of malignancy, features of Lambert-Eaton syndrome or other muscle changes. One patient had coeliac disease and symptoms resolved initially with a gluten free diet. When she represented with the physical symptoms of vertigo and fatigue, further improvement was seen with mycophenolate, resulting in return to study with no relapse for 2 years.

The time between onset of symptoms and initiation of immunosuppression in this treatment group ranged from 2 months to 2 years. There were no significant differences in the ‘red flag’ clinical features between these patients who responded to treatment and other patients in our psychiatric group, although this may be due to small sample size. Additionally, CSF examination did not necessarily discriminate between them.

However, a total of 19 other patients were offered immunotherapy without clear response. Of these, 14 were offered a disease-modifying anti-rheumatic drug (DMARDs) with or without corticosteroids and IVIg, one was offered glucocorticoids and IVIg, four offered minocycline only and four offered minocycline and another immunomodulatory agent (IVIg, hydroxychloroquine or corticosteroid).

Interestingly in our high cytokine group (at least one cytokine >4 standard deviations above the normal mean), n=3/6 patients responded to therapy whilst n=3/6 patients did not. Of the non-responders in this cohort, one was offered minocycline and then hydroxychloroquine but discontinued the medications within 2-3 months because of side effects and lack of perceived efficacy. One was treated with Plaquenil for 6 months with no clinical benefit seen. Mycophenolate was offered but the patient was lost to follow up. The third patient was treated with methotrexate, sirolimus and mycophenolate, with a duration of immunosuppression for greater than 12 months, with some modest improvements, but immunosuppression was ceased. She represented 2 years later with retinal vasculitis. Further investigations including a repeat lumbar puncture were recommended, but the patient was lost to follow up.

When CSF cytokines were compared across responders and non-responders, there was no significant difference between the two groups when examined with a Bonferroni adjusted p-value threshold of 0.002.

## Discussion

This, as an exploratory pilot study, sought to investigate the possibility of immune contribution to disease in a group of patients with complex, atypical or difficult to treat psychiatric disease. This is an area of increasing interest in psychiatry, particularly for patients with treatment resistance to standard treatments (having failed two or more agents) or for whom long term morbidity usually results in long term engagement with psychiatric services.

### Analysis of CSF cytokines studies suggest immune dysregulation in a subset of the psychiatric cohort

Our multivariable analysis identified two cytokines of particular interest. The interferon-γ–axis chemokine ITAC was elevated in the psychiatric cohort relative to NIND and AE controls. In contrast, the Th2-associated chemokine TARC was lower in the psychiatric cohort compared with NIND, AE, and VI groups in the random-effects model. Interpretation of the effect estimate of TARC is tempered by wide confidence intervals, reflecting limited precision due to sparse multinomial data and random-effects estimation. Nevertheless, the consistency and magnitude of the associations suggest that TARC may provide independent discriminatory information within a multivariable immune framework, despite the absence of significant univariate differences.

ITAC (CXCL11) is an interferon-γ–inducible chemokine that signals via the CXCR3 receptor (26) and pays a key role in Th1 polarised immune responses (27, 28). Microglia, the resident macrophages of the CNS express CXCR3 and respond to its ligands, including ITAC/CXCL11 (26). In the context of T-cell activation and polarization, CXCR3 receptor engagement leads to T-cell expression of IFN-γ (Th1 polarization) (27), consistent with the relative elevation of IFN-γ observed in our psychiatric cohort on univariate analysis. CXCR3 signalling also modulates activation of microglia (29, 30), which have been implicated in the CNS immune responses in infection (29, 31), autoimmunity (32) and in psychiatric disease (33). Increased levels of ITAC/CXCL11 are also associated with multiple autoimmune diseases (34–36) and in experimental autoimmune encephalomyelitis (EAE) (37). Together, these findings raise the possibility that dysregulation of the CXCL11–CXCR3 axis may contribute to altered T-cell polarisation and microglial activation in a subset of psychiatric presentations, a hypothesis that warrants confirmation in larger, longitudinal, and mechanistic studies.

TARC or CCL17 is classically associated with Th2 cells through the CCR4 receptor, mediating their recruitment to sites of atopic inflammation such as eczema (38). However, CCR4 is expressed many other cell types including T-regulatory cells. In oncological settings, tumour-derived CCL17 and CCL22 may recruit T-regulatory cells, contributing to local immune suppression (39, 40). Conversely, TARC/CCL17 suppresses Treg activity and promote atherosclerosis (41). Beyond Th2 cells and Tregs, TARC/CCL17 has chemotactic activity for other immune cells: monocytes, dendritic cells, natural killer (NK) cells, eosinophils and Th17 cells (42, 43). It has been implicated in inflammatory bowel disease (42), EAE, rheumatoid arthritis and osteoarthritis (43). In the latter two conditions, TARC/CCL17, alongside GMCSF, has been associated with pain phenotypes (43, 44). However, no significant difference in CSF TARC/CCL17 levels were observed between patients in our psychiatric cohort with and without chronic pain (p=0.14, z=-1.459), which may be due to limited statistical power.

Emerging data also suggest a CNS-specific role for TARC/CCL17. Murine models have reported increased TARC/CCL17 in the hippocampus and be associated with a reduction in activation of microglia and attenuation neuroinflammation (45, 46), whilst other models reported increased microglial activation but skewed towards the anti-inflammatory M2 phenotype (47). In humans, TARC/CCL17 has been reported to be increased in the plasma of patients with first episode psychosis (48), but reduced in the CSF (49) and brain tissue of (50) those with suicidal behaviour. Collectively, these findings may indicate that reduced CSF TARC in our psychiatric cohort may reflect a shift toward a more pro-inflammatory immune milieu, potentially characterised by diminished recruitment of regulatory immune subsets and relative dominance of interferon-γ–associated pathways.

The emergence of TARC as a significant factor only after adjustment for ITAC and batch effects supports the interpretation that TARC reflects relative immune balance rather than absolute cytokine abundance. In combination, elevated ITAC and reduced TARC may indicate a bias toward interferon-driven immune responses at the expense of regulatory or compartmentalising chemokine signals and/or perhaps antibody producing Th2 responses. These findings suggest that immune alterations in atypical psychiatric disease may be better characterised by shifts in immune activation pathway balance rather than isolated cytokine changes but again, this should be confirmed in a larger cohort.

The cytokine profile observed in our psychiatric cohort differs from much of the existing CSF cytokine literature in psychiatric disease, which is itself highly heterogeneous. IL-6 and IL-8 are the most frequently reported CSF cytokines associated with psychotic and mood disorders (51–62). Other reported CSF cytokines include IL-12β and IL-12p70 (57, 63), TNFα (57, 64), interferon-β (65), IL-4 (66), MCP-1 (67), and IL-1β in bipolar disorder and psychosis (51, 68–70). CSF TNFα has also been reported to be increased in depression (59). However, some studies also reported no differences between patients with psychiatric disease and controls (64, 66, 71–73), decreased CSF IL-6 in patients with schizophrenia (67, 74) or elevation only in IL-6 but not in IL-8 (60). Methodological heterogeneity across studies: including differences in psychiatric phenotypes, cytokine panels, assay platforms, sensitivity thresholds, and control groups, likely contributes to these inconsistencies. Control cohorts have varied widely, including healthy volunteers (61, 64, 71, 74, 75), non-inflammatory neurological controls (72), and patients with other neurological diseases (67, 73), complicating direct comparisons.

Most of the current literature has examined cohorts with well-defined psychiatric diagnoses. Importantly, our cohort of psychiatric patients was defined by having atypical disease and the presence of clinical red flag features that required further assessment to exclude an underlying cause such as autoimmunity. As such, this population may represent a biologically distinct subgroup with different dominant pathophysiological mechanisms. Additionally, results may differ because of differences in the sensitivities of the assays used. Hence the finding of a different cytokine profile here is not unexpected.

### A subset of our patients had high CSF cytokines

A substantial subset of patients in our psychiatric cohort expressed markedly higher CSF cytokines, with six individuals having at least one CSF cytokine greater than 4 standard deviations above the normal mean, consistent with pronounced immune activation. The pattern of cytokine elevation was heterogenous amongst this group, whilst routinely available CSF markers of neuroinflammation were mostly normal, except for neopterin. This heterogeneity of response can also be seen in autoimmune encephalitis, where patients may exhibit elevation in only a limited number, or none, of the many routinely available surrogate markers of neuroinflammation.

Although interpretation is constrained by the modest sample size and clinical heterogeneity of the cohort, these findings underscore the potential value of CSF cytokine profiling in patients with atypical psychiatric presentations and clinical “red flag” features for further investigation of pathogenic pathways. In addition, in the context of completely normal traditional CSF indices, some patients with a suspicious clinical phenotype still respond to a trial of immunosuppression. Together, these observations highlight the need for more sensitive and discriminative biomarkers of neuroinflammation to guide investigation and management when clinical suspicion remains high.

### Conventional markers of neuroinflammation can be normal

Most of our psychiatric disease cohort had normal conventional CSF parameters. MRI was also considered largely normal in all patients.

There are studies in the literature that report increased markers of immune activation in psychiatric patients. Elevation in serum markers of acute phase reactants indicating inflammation (76–79) or proinflammatory serum cytokines have been reported (80–83). Disturbances of the blood-brain barrier, alterations in CSF protein levels, CSF pleocytosis, and the presence of oligoclonal bands have also been documented (58, 84–92), although there are reports that conflict with these findings (58).

Even in our AE cohort (22), only a minority of patients had positive biomarkers for neuroinflammation in CSF. This known lack of sensitivity of currently available investigations highlights the need for better biomarkers for the detection of autoimmune contributions to psychiatric disease.

#### Evaluation of immune parameters in atypical psychiatric disease is worthwhile

Currently, the diagnostic criteria for autoimmune encephalitis include an acute or ‘subacute onset of symptoms’ (6). However, recognition of autoimmune contributions to psychiatric illness is relatively recent, and patients with treatment-resistant psychiatric disease may present to immunology or neurology services many years after symptom onset, following multiple trials of psychotropic therapies. In this context, accurate assessment of the timing and rapidity of initial symptom onset may be difficult. Delays in presentation are common in psychiatric disorders due to poor symptom recognition, social stigma, and impaired insight. Additionally, the cognitive difficulties often associated with immune contribution to psychiatric disease further limit reliable reconstruction of early disease features.

In our psychiatric cohort, six patients (17%) had their diagnosis revised based on clinical features, routinely available diagnostic investigations, and response to immunosuppressive therapy, indicating an underlying autoimmune disorder (**Table 7**). All demonstrated clinical improvement following disease-specific treatment, with reduction or cessation of psychotropic medications.

These cases illustrates the importance of early clinical suspicion and periodic reassessment when atypical features or recognised “red flags” are present, as diagnostic reconsideration may significantly alter management and outcomes, particularly delay in treatment in patients with autoimmune encephalitis increases the risk of long-term sequalae (93). In our cohort, at least six patients with long-standing treatment-resistant disease experienced substantial resolution of psychiatric symptoms following appropriate immune-directed therapy, and three no longer required psychotropic medication. This underscores the importance of comprehensive evaluation and diagnostic reappraisal in patients with refractory psychiatric illness, particularly when clinical features raise concern for an autoimmune process.

Diagnostic revision following CSF analysis has also been reported elsewhere. In a systematic review, two studies identified diagnostic changes in 3.2% and 6% of psychiatric cases, respectively (58). While our cohort represented a selected high-risk population, immune involvement has also been demonstrated in broader psychiatric samples. In a recent multicentre study of 1,114 individuals enrolled in first-episode schizophrenia trials, 3.7% were positive for anti-NMDA receptor antibodies; these patients exhibited better overall functioning and fewer negative symptoms, but were otherwise clinically similar to antibody-negative participants (94). Another recent study reported 8% of their psychiatric disease cohort had diagnosis revised to a neurological disorder after CSF analysis (92).

There is increasing recognition that psychotropic medications exert immunomodulatory effects (95–108). At the same time, growing evidence supports the use of immune-modulating therapies in selected psychiatric populations, ranging from non-steroidal anti-inflammatory drugs (109, 110) to biologics such as canakinumab (111) or rituximab (112). These developments further support the potential utility of immune profiling in patients with atypical or difficult-to-treat psychiatric disease.

Importantly, where an alternative autoimmune diagnosis was established in our cohort, response to immunosuppressive therapy was observed even years after symptom onset (113). This suggests that patients with prolonged, refractory psychiatric illness may still benefit from reassessment and immune-directed treatment when clinical suspicion remains high.

### Diagnostic results must be considered in their clinical context

Despite the promising results in a subset of our cohort, careful interpretation of both clinical and investigative findings remains essential when re-evaluating patients. Diagnostic conclusions should not be based on isolated symptoms or a single test result. Many patients in our psychiatric cohort reported additional physical symptoms including fatigue, cognitive disturbance, seizures or seizure like episodes, sleep disturbance, paraesthesia and chronic pain (**Table 2**). Whilst these symptoms may raise suspicion for autoimmune driven disease, they are also common in psychiatric disorders and may be attributable to psychotropic medication use (114–120). The emergence of new physical symptoms or their predominance in the clinical picture should prompt reassessment, but their significance must be interpreted in the context of the broader clinical and investigational findings.

Results of autoimmune serology likewise require cautious interpretation within the clinical context. The presence of an autoantibody in the absence of compatible clinical features may indicate autoimmune susceptibility rather than active disease. However, the clinical spectrum of autoantibody-mediated conditions continues to expand, necessitating a more nuanced approach. Interpretation is further complicated by limited understanding of epitope specificity and uncertainty regarding whether detected antibodies are pathogenic drivers or secondary phenomena, such as markers of neurodegeneration. Our patient with positive VGCC antibodies in the serum had no features of associated muscle changes or evidence of malignancy, rendering their clinical relevance uncertain, although she demonstrated improvement with immunosuppression. Notably, an association between VGCC antibodies and schizophrenia has been reported (121). Another patient had borderline myositis-associated antibodies without clinical evidence of myositis or interstitial lung disease, again of unclear significance. With the increasing discovery of relevant autoantibodies to CNS disease, and the recognition of dual antibody conditions (122–124), the presence of other yet to be defined autoantibodies may be in play.

Low to mid-titre ANA positivity was common in our patient cohort, but this finding has also been reported (albeit at lower prevalence) in otherwise healthy individuals (125). Most ANA-positive patients lacked clinical features of connective tissue disease or associated ENA or dsDNA positivity. One patient with low-titre ANA was trialled on immunosuppression due to treatment resistance and a recent history of ovarian cancer but showed minimal clinical improvement. While ANA testing is highly sensitive for autoimmune disease, it lacks specificity and must be interpreted alongside clinical features and more disease-specific antibodies. Higher ANA titres (>1:640) occur in up to 2.5% of the general population (126) and may precede overt autoimmune disease by many years. Similarly, myositis-associated antibodies have high false-positive rates (127, 128), limiting their diagnostic utility in the absence of characteristic clinical features.

Thyroid autoantibodies were the second most common serological finding, with up to 20% of patients positive for thyroid peroxidase antibodies. While these antibodies are associated with Hashimoto’s encephalopathy, they are also present in up to 11% of the general population (129) and cannot in themselves be used to confirm a diagnosis of thyroid disease (130). Additionally, Hashimoto’s encephalopathy remains diagnostically controversial. It is thought to be rare, with heterogenous presentations, and has no specific biomarkers. In some large cohorts of suspected Hashimoto’s encephalopathy, the majority of patients were subsequently found to have a non-immune mediated diagnosis (131). Although steroid responsiveness has traditionally been considered supportive, only 31% of patients in a recent series fitting the diagnostic criteria achieved complete response with corticosteroids alone, often requiring additional immunosuppression (132), consistent with experience at our centre.

In patients with severe, treatment-resistant psychiatric illness, a therapeutic trial of immunosuppression may be considered. However, current evidence supports such an approach only when there is demonstrable immune-mediated pathology, ideally guided by inflammatory biomarkers, and after a thorough risk–benefit assessment (6, 133). Meanwhile, the rapidly evolving understanding of immune-mediated disease, the identification of novel autoantibodies, the presence of CSF cytokine abnormalities despite normal conventional indices, and evidence that delayed immunotherapy may worsen outcomes raise the question of whether, in selected cases, withholding a carefully monitored, informed-consent trial may itself carry risk.

Immunosuppressive regimens inherently entail risks and side effects. In our cohort, combined immunotherapy and psychiatric treatment did not result in a clear increase in adverse events compared with psychotropic therapy alone (113), although this study was not powered to definitively assess safety. In the absence of conclusive evidence for or against immunotherapy in this population, treatment decisions should be made within a multidisciplinary framework, with patients or their guardians: fully informed of the available evidence, uncertainties, risks, and potential benefits. Given the high likelihood of lifelong morbidity and social exclusion in refractory psychiatric disease, we also advocate for rigorous outcome monitoring and systematic biospecimen collection to facilitate validation of disease-associated biomarkers and support future therapeutic advances in this challenging patient group.

### Study limitations

Our study is limited by our relatively small and heterogenous cohorts in which not all results were available for all patients. This is in the context of there being no clear standard of investigations indicated for these patient groups. There is a referral bias in our study groups, implicit in our recruitment method and our comparison cohorts were derived from previous cohorts recruited for a study in our centre (22). Numbers in our comparison cohorts were also small. In addition, although the recent consensus criteria emphasise subacute onset as a key feature of autoimmune-related psychiatric presentations (21), many patients were assessed years after symptom onset. Delayed evaluation, often compounded by cognitive impairment, poor symptom recall, and barriers to early psychiatric care, may have limited our ability to accurately characterise disease onset. Nevertheless, all included patients had at least one ‘red flag’ feature in their clinical presentation giving indication of a need for further evaluation.

Methodological limitations related to CSF sampling should also be considered. A total CSF volume of 10–15 mL was collected to support comprehensive neurological testing, exceeding volumes typically required for routine CSF analyses (1-2mL). However, not all samples were collected in RPMI medium, limiting the availability and interpretability of CSF flow cytometry data in some patients (134). Serum cytokine levels were not measured, precluding assessment of the relationship between circulating and intrathecal cytokine profiles; this represents an important direction for future studies.

As an exploratory pilot study, our findings require validation in larger cohorts. The heterogeneity of atypical, treatment-resistant psychiatric disease would ideally be addressed through clustering or subgroup analyses; however, our sample size constraints precluded such approaches. Similarly, while psychotropic medication class was considered in our analyses, larger studies would be required to examine the effects of dosage, treatment duration, and medication combinations.

The choice of control groups represents an additional limitation. Ethical constraints precluded CSF sampling from healthy volunteers, necessitating the use of neurological control populations traditionally considered non-inflammatory. Emerging evidence suggests immune involvement in some of these “non-inflammatory” conditions (135, 136), which may have attenuated observed differences between groups. Comparison with truly healthy age and sex matched CSF controls would be preferable where feasible.

Our psychiatric cohort demonstrated a female predominance, consistent with many autoantibody-associated autoimmune diseases (137) and most antibody-positive AE (138). In contrast, antibody-negative AE is reported to have a 1:1 male:female gender divide (139), or in the case of our previously reported cohort, a male predominance (22). These differences may reflect sex-specific immune mechanisms, underscoring the need to examine psychiatric populations to compare cytokine signatures directly across both typical and atypical diagnostic groups to better define the sensitivity and specificity of current selection criteria.

Due to chronicity of disease, symptoms in our patients may be due to irreversible CNS injury rather than ongoing immune activity and investigations may be impacted by various psychotropic drugs (140, 141). However, without appropriate investigations, the possibility of immune contribution cannot be excluded in these treatment resistant patients.

## Conclusion

Our study demonstrates the clinical use of a large and comprehensive panel of immunological investigations in a psychiatric patient cohort selected based on “red flags” or treatment resistance which may identify patients where an alternate diagnosis should be pursued. This study highlights the need for recurrent re-evaluating in patients with difficult-to-treat or atypical presentations of psychiatric illness to avoid diagnostic anchoring. However, in reevaluating patients, the clinical scenario must be used with investigation results to consider whether an immune-mediated disease process is possible and may be causative rather than simply an association, and whether directed immune modulatory therapy may provide benefit.

Basic questions remain about the general role of autoimmunity and inflammation in the pathogenesis of psychiatric illness. Therefore, clinical boundaries of the target group of people with a psychiatric illness who should be referred for this intensive work-up or circumstances when a trial of immunotherapy should be considered remain unclear. However, while not formally investigated, from a health economic view, any cost saving in care requirements from significantly improved function of patients responding to immunomodulation may be significantly higher than the costs of investigations for the whole group. Further study of these patients may yield a better understanding of both diagnostic algorithms to identify patients with immune contribution to disease and targets for treatment.

## Data Availability

The raw data supporting the conclusions of this article will be made available by the authors, without undue reservation.

## Glossary

AE: Autoimmune encephalitis
ANA: Anti-nuclear antibody
ANCA: Anti-neutrophil cytoplasmic antibody
BBB: Blood–brain barrier
BCA-1: B-cell–attracting chemokine 1 (CXCL13)
C3/C4: Complement component 3/4
CCR4: C-C chemokine receptor type 4
CD4/CD8: Cluster of differentiation 4/8
CNS: Central nervous system
CSF: Cerebrospinal fluid
CXCL: C-X-C motif chemokine ligand
CXCR: C-X-C chemokine receptor
DMARDs: Disease-modifying anti-rheumatic drugs
dsDNA: Double-stranded DNA
EEG: Electroencephalography
ELISA: Enzyme-linked immunosorbent assay
ENA: Extractable nuclear antigens
ENTV: Enterovirus
EPG/IFE: Electrophoresis/Immunofixation electrophoresis
ESR: Erythrocyte sedimentation rate
EAE: Experimental autoimmune encephalomyelitis
GAD: Glutamic acid decarboxylase
G-CSF: Granulocyte colony-stimulating factor
GFAP: Glial fibrillary acidic protein
GM-CSF: Granulocyte-macrophage colony-stimulating factor
HSV: Herpes simplex virus
IFN-γ: Interferon gamma
IgA/IgG/IgM: Immunoglobulin A/G/M
IIH: Idiopathic intracranial hypertension
IL: Interleukin
IP-10: Interferon-gamma–induced protein 10 (CXCL10)
ITAC: Interferon-inducible T-cell alpha chemoattractant (CXCL11)
IVIg: Intravenous immunoglobulin
LEMS: Lambert–Eaton myasthenic syndrome
MHC I: Major histocompatibility complex class I
MPO: Myeloperoxidase
MRI: Magnetic resonance imaging
NIND: Non-inflammatory neurological disease
NK/NKT: Natural killer/natural killer T cells
NPH: Normal pressure hydrocephalus
NSAIDs: Non-steroidal anti-inflammatory drugs
PCR: Polymerase chain reaction
PCA: Principal component analysis
PR3: Proteinase 3
RBC: Red blood cells
RPMI: Roswell Park Memorial Institute
RRR: Relative risk ratio
Th1/Th2/Th17: T-helper cell subtype 1/2/17
TNF-α: Tumour necrosis factor alpha
TARC: Thymus and activation-regulated chemokine (CCL17)
VGCC: Voltage-gated calcium channel
VGKC: Voltage-gated potassium channel
VI: Viral infection
VZV: Varicella zoster virus
β2-microglobulin: Beta-2 microglobulin
