# Supplementary material for: Markers of neuroinflammation in the CSF of patients with difficult to treat psychiatric disease
Source: Front Psychiatry. 2026 May 4;17:1665447. doi: 10.3389/fpsyt.2026.1665447 (PMC13180878; doi:10.3389/fpsyt.2026.1665447)
Supplement: Supplementary file 1 [file SupplementaryFile1.docx]

**Supplementary Methods**

All investigations, unless otherwise stated, were performed at New South Wales Health Pathology’s Institute for Clinical Pathology and Medical Research (NSW Health Pathology- ICPMR-Westmead, Australia). Patients underwent conventional investigations for AE including blood tests and lumbar puncture for collection of CSF.

Conventional serum and CSF studies were: isoelectric focussing for oligoclonal bands (Sebia Paris, France), neopterin (in house HPLC assay), indirect immunofluorescence (IIF) on primate brain (Inova San Diego, USA) and line blot (PCA-1, PCA-2, ANNA-1, ANNA-2, Ma-1, Ma2, Amphiphysin, CV2, CRMP5) for onconeural antibodies (Ravo Bettlach Switzerland), as well as a limbic encephalitis panel (NMDAR, LGI-1, CASPR2, GABA(B), DPPX, IgLON5) on HEK2 transfected cells (fixed cell-based assay, Euroimmun Lubeck, Germany).

Additionally, CSF studies included: CSF: microscopy; culture; and protein (Siemens Vista Erlangen, Germany), anti-glutamic acid decarboxylase (GAD) antibodies (ELISA, SEALS Pathology, Prince of Wales Hospital, NSW Australia; RSR Cardiff, UK) and polymerase chain reaction (PCR) for viral infections: HSV (Artus Hamburg, Germany), VZV (in-house PCR assay), ENTV (in house PCR assay) and EBV by PCR (Ellitech Paris, France) were run.

The following routinely available diagnostic tests were also performed on serum on patients with psychiatric disease: anti-thyroid antibody (Siemens Munich, Germany and Abbott Architect Illinois, USA) and thyroglobulin antibody (Siemens Munich, Germany and Abbot Architect Illinois, USA); ANA (Euroimmun Lubeck, Germany), ENA (Orgenteck ELISA), dsDNA (Bioflash Werfen Barcelona, Spain), coeliac autoantibodies (ELISA and indirect immunofluorescence (for anti-endomysial antibodies), Inova San Diego, USA), EPG/IFE (Sebia, Lisses, France), ESR (Starrsed), liver autoantibodies by indirect immunofluorescence (multiblock) on rat liver kidney stomach tissue substrate (Aesku), anti-GAD antibodies(RSR ELISA), anti IA2 (RSA ELISA), and anti-ZNT8 antibodies (Euroimmun), IgG, IgA, IgM (Abbot Architect,), C3, C4 (Abbot Architect Illinois, USA), IgG subclasses (Siemens BNII, Erlangen Germany), as well as voltage-gated potassium antibodies (VGKC) and voltage gated calcium channel antibodies (VGCC) radioimmunoassay; Queensland Pathology, Royal Brisbane Hospital, Australia (kits from RSR Cardiff, United Kingdom)

CSF examination by flow cytometry used CD19 (BV421) and CD20 (APC-AF750) to enumerate B cells, CD3(PE-CF594) for T cells along with CD4 (FITC) and CD8 (PE) for CD4:8 ratios. CD3-CD4+ was used as a surrogate marker for monocytes.

All conventional investigations were collected according to current practice and performed according to the usual procedures available at the receiving diagnostic laboratory.

A broad range of CSF cytokines were performed using a bead-based multiplex luminex assay on a research base only (Milliplex; Merck Millipore Darmstadt Germany; Table e-1) using the magnetic multi bead array kits (MPHSCTMAG28SK17; MPHCYP3MAG63K0; MPHCYTOMAG60K02; MPHCYP2MAG62K01). Cytokines tested were: IFN-γ, ITAC (CXCL11), IL-12p70, TNF-alpha, CXCL9, CXCL10/IP-10, IL-13, IL-4, IL-5, TARC (CCL17), Eotaxin, IL-17a, IL-6, IL-8, IL-1b, IL-21, IL-2, IL-23, IL-7, IL-10, BCA-1(CXCL13), GMCSF and GCSF. CSF cytokines were run by two operators and in duplicate except when the sample amount was insufficient when it was run in singlicate.
